# Epidemiology of trauma deaths across a mature regional trauma system: patterns of prehospital and in-hospital fatalities

**DOI:** 10.1093/bjs/znag030

**Published:** 2026-03-18

**Authors:** Aditi Nijhawan, Ewoud ter Avest, Callum J Twohig, Stacey J Webster, Jared M Wohlgemut, Jason Morris, Robbie Lendrum, Virginia Fitzpatrick-Swallow, David J Lockey, Zane B Perkins

**Affiliations:** London’s Air Ambulance, London, UK; Major Trauma Service, Royal London Hospital, Barts Health NHS Trust, London, UK; London’s Air Ambulance, London, UK; Major Trauma Service, Royal London Hospital, Barts Health NHS Trust, London, UK; Department of Acute Care, University Medical Center Groningen, Groningen, The Netherlands; London’s Air Ambulance, London, UK; Centre for Trauma Sciences, Queen Mary University of London, London, UK; London’s Air Ambulance, London, UK; Academic Department of Military Emergency Medicine, Royal Centre for Defence Medicine (Research & Clinical Innovation), Birmingham, UK; Centre for Trauma Sciences, Queen Mary University of London, London, UK; London’s Air Ambulance, London, UK; London Ambulance Service NHS Trust, London, UK; London’s Air Ambulance, London, UK; Major Trauma Service, Royal London Hospital, Barts Health NHS Trust, London, UK; Home Office Registered Forensic Pathologist, Wantage, UK; London’s Air Ambulance, London, UK; Major Trauma Service, Royal London Hospital, Barts Health NHS Trust, London, UK; Centre for Trauma Sciences, Queen Mary University of London, London, UK; London’s Air Ambulance, London, UK; Major Trauma Service, Royal London Hospital, Barts Health NHS Trust, London, UK; Centre for Trauma Sciences, Queen Mary University of London, London, UK

## Abstract

**Introduction:**

In mature trauma systems, most trauma deaths occur soon after injury yet studies often only include patients who survive to hospital admission. These studies exclude prehospital deaths and introduce substantial survival bias. Understanding trauma fatalities across all phases of care is essential to identify opportunities to further improve trauma outcomes.

**Methods:**

This retrospective cohort study analysed adult trauma fatalities attended by London’s Air Ambulance from 1 January 2019 to 31 December 2020. Deaths were classified as prehospital if traumatic cardiac arrest occurred before hospital arrival. Timing of death was recorded as minutes from injury to arrest for prehospital cases and days from admission to death for in-hospital cases. A multidisciplinary panel determined the likely cause of death using clinical, radiological, and post-mortem findings.

**Results:**

Among 3089 adult trauma patients attended, 497 (16.1%) died. Most deaths (77.1%) occurred prehospital, with a median time from injury to traumatic cardiac arrest of 12 min (i.q.r. 6–24). Haemorrhage and traumatic brain injury accounted for 81.9% of deaths overall, but their distribution differed markedly by phase of care: 96.2% of haemorrhage deaths occurred prehospital, whereas 84.2% of in-hospital deaths were due to traumatic brain injury. In addition, deaths from all other potentially reversible causes, and 95.1% of penetrating trauma deaths, occurred prehospital.

**Conclusion:**

In mature trauma systems, most trauma deaths now occur before arrival in hospital, with many due to potentially reversible causes. The greatest opportunities to improve trauma outcomes now lie in the prehospital phase of care.

## Teaser

Serious injury is a major cause of death, especially in younger people. Although hospital trauma care has improved, most people who die after serious injury die before reaching hospital. This study examined when and why adult trauma patients died within a well-developed, organized urban trauma system. Most deaths occurred very soon after injury and usually before hospital admission, often due to severe bleeding or other conditions that can usually be treated if care is given quickly in hospital. In contrast, patients who reached hospital rarely died from these potentially treatable problems. These findings show that further reductions in trauma deaths are most likely to come from action before hospital arrival. This includes preventing violent injury, helping members of the public and first responders to control life-threatening bleeding, and ensuring access to life-saving treatments before hospital arrival, alongside rapid transport to hospital.

## Introduction

Trauma is a global public health problem, responsible for one in ten deaths and the leading cause of years of life lost worldwide^1^. In mature trauma systems, most fatalities occur soon after injury, forming a consistent unimodal ‘curve of death’^2,3^. However, most outcome studies focus on patients who survive to hospital admission, overlooking prehospital deaths and generating significant survival bias. Understanding the cause and timing of trauma deaths across all phases of care is essential to guide system development, set research priorities, and identify where further improvements in survival can be achieved^4^.

The temporal distribution and causes of trauma deaths were first characterized by Baker and Trunkey in their seminal population-based analysis of fatalities in San Francisco^5,6^. They described a ‘trimodal’ distribution, with immediate deaths at the scene, early hospital deaths within hours of injury largely due to haemorrhage or brain injury, and a later peak from multiple organ failure. This work fundamentally shaped modern trauma care, directly informing the design of organized trauma systems. Subsequent population-based studies demonstrated the impact of these reforms: advances in trauma care produced a marked left shift in fatality distribution—the late peak largely disappeared, early deaths occurred sooner, and a greater proportion of fatalities occurred before hospital arrival^7–10^. Across these series, central nervous system injury and haemorrhage remained the predominant causes of death, together accounting for approximately 70–80% of fatalities^7–10^. More recent civilian and military analyses have confirmed this highly reproducible, left-shifted unimodal pattern, described by Holcomb and colleagues as the ‘consistent curve of death’, and emphasized that further improvements in survival will depend on advances in prehospital care^2^.

Population-based studies of trauma death are rare, but those from other high-income regions also report a left-shifted unimodal distribution dominated by early, prehospital deaths^11–13^. In the UK, an inclusive regional trauma system was established in 2012, leading to major improvements in the organization and delivery of trauma care^14^. These reforms improved access to specialist centres and the timeliness of life-saving interventions, with in-hospital death from haemorrhage almost halved over the following decade^15^. Yet, as with most trauma outcome studies, these analyses were limited to hospital data and therefore could not assess deaths occurring before arrival. As hospital survival continues to improve, understanding the causes and timing of prehospital deaths has become a key priority for further reducing preventable deaths in mature trauma systems.

The current study aimed to describe the timing, setting, and causes of major trauma deaths within a mature regional trauma system, including those occurring during the prehospital phase of care.

## Methods

### Study design

This retrospective cohort study analysed all trauma fatalities among patients attended by London Air Ambulance (LAA) between 1 January 2019 and 31 December 2020. The project was classified as a service evaluation by Barts Health NHS Trust and did not require research ethics committee review. Use of deidentified outcome data was approved by the institutional Clinical Effectiveness Unit (registration number: 12778). The study is reported in accordance with the STROBE guidelines for observational studies^16^.

### Setting

The London Trauma System is an inclusive regional trauma network serving approximately 10 million residents of Greater London, integrating the London Ambulance Service (LAS), LAA, four major trauma centres, and 22 trauma units. LAA provides a 24/7 physician–paramedic service and operates as part of the LAS response to suspected major trauma across the region. LAA is dispatched to approximately 93–95% of major trauma cases in London, as defined by the London Major Trauma Triage Decision Tool 2020^17^, with an average dispatch miss rate of 0.4% and an operational miss rate of 6.0%.

### Study population

All adult trauma patients (aged ≥16 years) attended by LAA who subsequently died were included. Deaths following hanging, drowning, fire, or electrocution were excluded.

### Data sources

Prehospital deaths were identified through manual review of all LAA patient records. In-hospital outcome data were obtained from the UK Trauma Audit and Research Network, the national trauma registry, from which records for London trauma admissions were extracted. Additional information was obtained from ambulance dispatch records, hospital records and imaging, coroner’s reports, and post-mortem examinations. These sources informed determination of the timing of traumatic cardiac arrest and the likely cause of death, which was ascribed by a multidisciplinary panel of consultants in prehospital care, trauma surgery, and forensic pathology at a dedicated monthly Clinicopathological Correlation meeting.

### Definitions

Deaths were classified as prehospital if traumatic cardiac arrest (TCA) occurred before admission, and as in-hospital if death occurred after admission without prior TCA. TCA was defined as loss of consciousness with absent or agonal respiration and an absent central pulse following traumatic injury. For prehospital cases, timing of death was measured in minutes from injury to TCA; for in-hospital cases, in days from admission to death. Time of injury was defined as the earliest reliable documented time, usually the emergency (‘999’) call time, unless a more precise witnessed injury time was available. Deaths due to airway obstruction, traumatic asphyxia, or tension pneumothorax were grouped as ventilatory failure. Injuries were classified as *incompatible with life* according to the JRCALC Recognition of Life Extinct (ROLE) criteria.

### Outcomes

The primary outcomes were the timing of death and the ascribed cause of death.

### Statistical analysis

Data analyses were performed using GraphPad Prism, version 9 (GraphPad, La Jolla, CA). Normality was assessed with histograms and the Shapiro–Wilk test. Continuous variables are reported as median with interquartile range, and categorical variables as counts and percentages. Group comparisons used the Mann–Whitney U, χ^2^, or Fisher’s exact tests, as appropriate. No imputation was performed for missing data. A two-sided *P* < 0.05 was considered statistically significant.

## Results

During the two-year study period, LAA attended 3089 patients, of whom 497 (16.1%) died. Demographic and injury characteristics are summarized in *Table 1*. Most deaths occurred prehospital (383, 77.1%), with 114 (22.9%) occurring after hospital admission. Most deaths occurred on the day of injury (398, 80.1%) with median time from injury to TCA 12 min (i.q.r. 6–24) among prehospital deaths. In hospitalized patients, median time from admission to death was 2 days (i.q.r. 1–6; range 0–87) (*Fig. 1*).

**Fig. 1:**
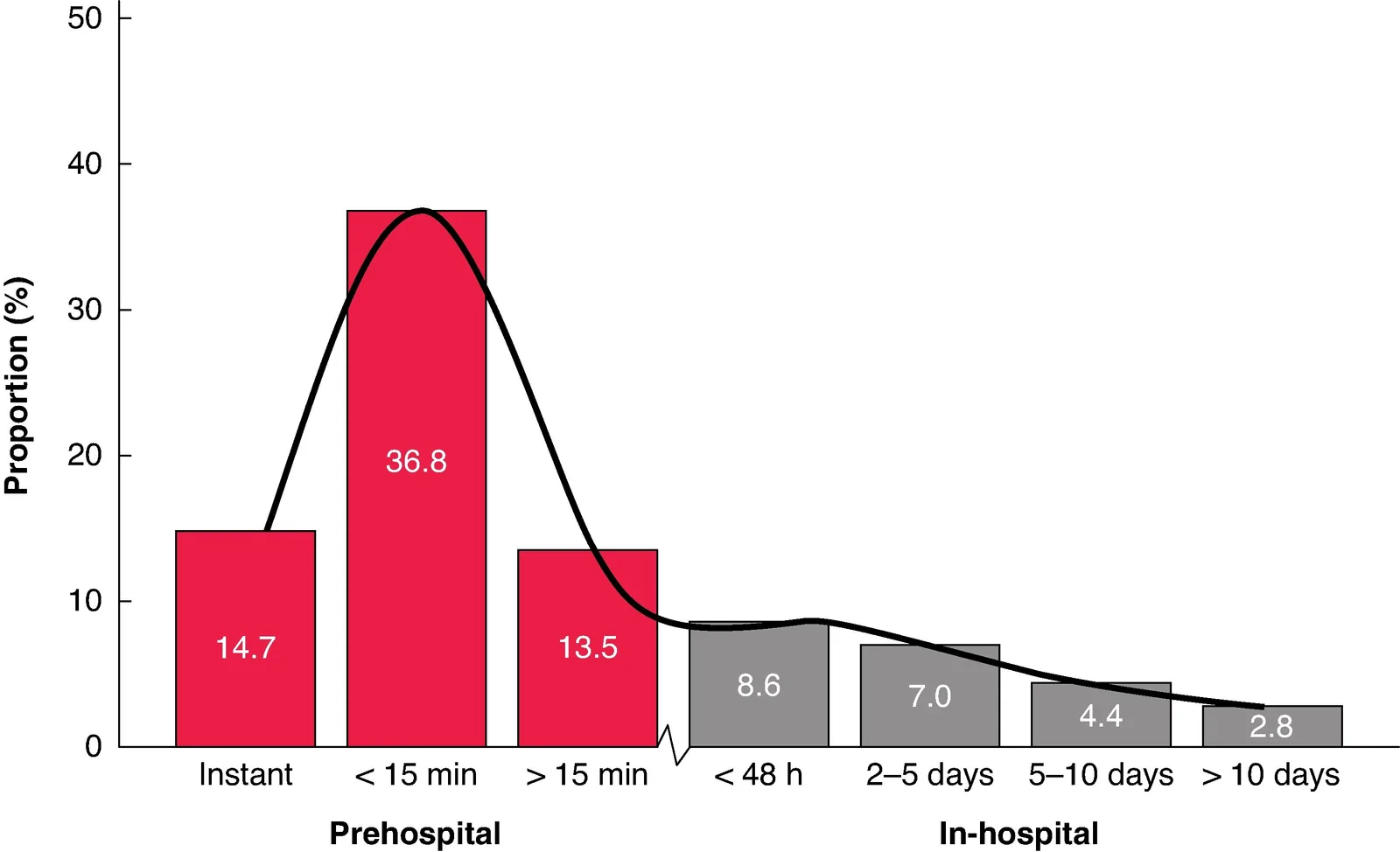
Timing of trauma deaths in a mature trauma system.

Prehospital deaths are shown as time from injury to traumatic cardiac arrest (in minutes); in-hospital deaths are shown as time from admission to death (in days). Red bars represent prehospital deaths; grey bars represent in-hospital deaths. For 60 cases (15.6%), the exact time of death could not be established; all were prehospital deaths. Percentages therefore do not sum to 100%.

**Table 1:** Baseline characteristics of 497 trauma deaths by setting (prehospital *versus* in-hospital)

		Trauma deaths			
Characteristic	Missing data (%)	Overall (*n* = 497)	Prehospital (*n* = 383)	In-hospital (*n* = 114)	*P*
Age, years	40 (8.1)	35 (25–52)	31 (25–49)	51 (30–71)	<0.0001
Male	2 (0.4)	400 (80.8)	316 (82.9)	84 (73.7)	0.031
**Mechanism of injury**					
Penetrating	0	144 (29.0)	137 (35.8)	7 (6.1)	<0.0001
Non-ballistic		125 (25.2)	119 (86.7)	6 (85.7)	1.0
Ballistic		19 (3.8)	18 (13.1)	1 (14.3)	1.0
**Blunt**	0	353 (71.0)	246 (64.2)	107 (93.9)	<0.0001
RTC—motor vehicle		22 (4.4)	13 (3.4)	9 (7.9)	0.337
RTC—motorcycle		40 (8.1)	32 (8.4)	8 (7.0)	0.147
RTC—cyclist		12 (2.4)	7 (1.8)	5 (4.4)	0.523
RTC—pedestrian		79 (15.9)	50 (13.1)	29 (25.4)	0.167
Person hit by train		56 (11.3)	55 (14.4)	1 (0.9)	<0.0001
Fall from height		91 (18.3)	74 (19.3)	17 (14.9)	0.005
Fall down stairs		29 (5.8)	4 (1.0)	25 (21.9)	<0.0001
Fall from standing		9 (1.8)	2 (0.5)	7 (6.1)	0.004
Assault		8 (1.6)	3 (0.8)	5 (4.4)	0.058
Other		7 (1.4)	6 (1.6)	1 (0.9)	0.680
**Cause of death**					
Ventilatory failure*		16 (3.2)	16 (4.2)	0	0.029
Cardiac tamponade		11 (2.2)	11 (2.9)	0	0.077
Haemorrhage		183 (36.8)	176 (46.0)	7 (6.1)	<0.0001
CNS injury		194 (39.0)	98 (25.6)	96 (84.2)	<0.0001
Multiple causes		30 (6.0)	30 (7.8)	0	0.001
Multiorgan failure		8 (1.6)	0	8 (7.0)	<0.0001
Injuries incompatible with life†		37 (7.4)	37 (9.7)	0	<0.0001
Other/unclear		18 (3.6)	15 (3.9)	3 (2.6)	0.775

Data are presented as number (%) or median (i.q.r.). RTC, road traffic collision; CNS, central nervous system. *P* refers to comparisons between prehospital and in-hospital groups. *Ventilatory failure includes deaths from airway obstruction, traumatic asphyxia, and tension pneumothorax. †Injuries incompatible with life were defined according to the JRCALC Recognition of Life Extinct (ROLE) criteria (for example decapitation, massive cranial destruction, hemicorporectomy, or similar massive injuries).

The likely cause of death was determined for 482 patients (97.0%) through multidisciplinary case review. In 333 cases (67.0%), this determination was supported by radiological, operative, or post-mortem findings (*Tables S1*, *S2*). The most frequently ascribed causes of death were central nervous system (CNS) injury (194, 39.0%), haemorrhage (183, 36.8%), or a combination of both (30, 6.0%) (*Fig. 2a*). Marked differences were observed between prehospital and in-hospital deaths (*Fig. 2b*): all deaths from airway obstruction, traumatic asphyxia, tension pneumothorax, and cardiac tamponade occurred prehospital. Only 7 of 183 deaths from haemorrhage occurred in patients admitted to hospital (3.8%). In-hospital deaths were predominantly due to CNS injury (96 of 114, 84.2%).

**Fig. 2:**
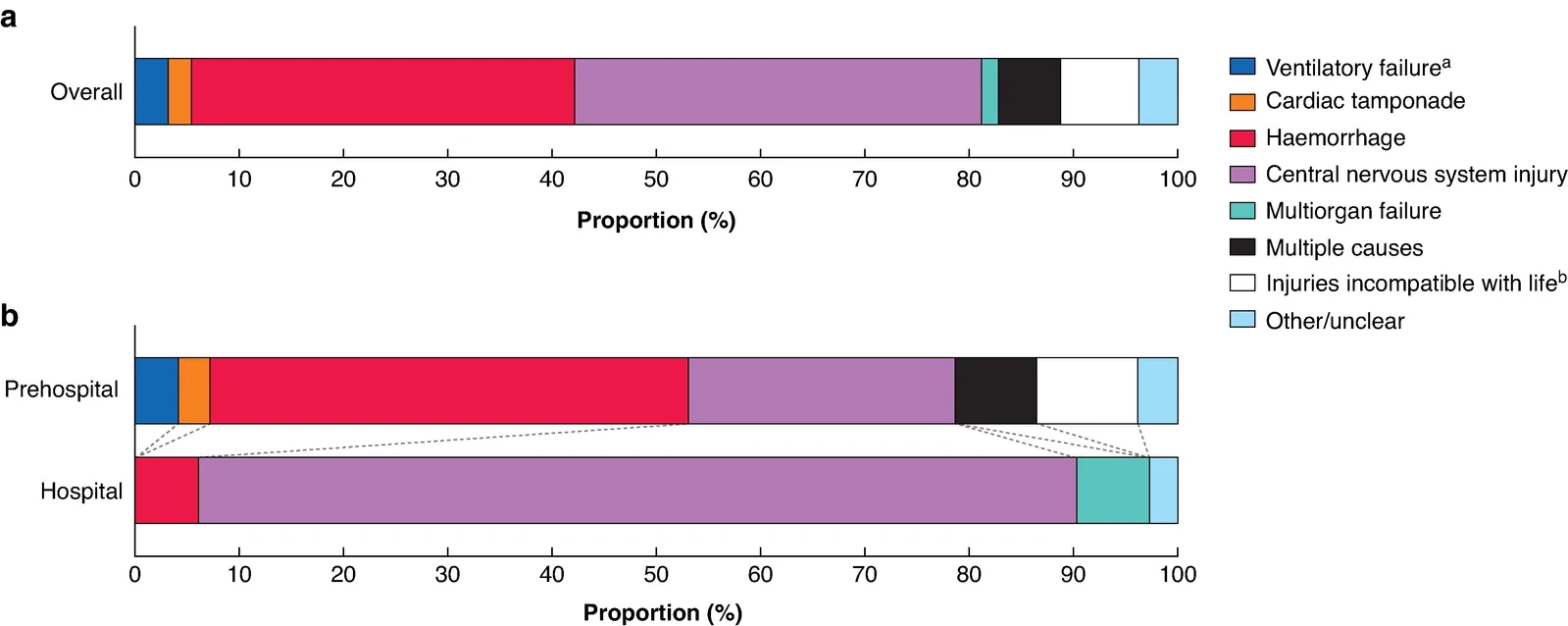
Causes of trauma deaths in a mature trauma system.

**a** Distribution of likely causes of death across the whole cohort. **b** Distribution of likely causes of death stratified by phase of care (prehospital *versus* in-hospital). Likely causes of death were determined by a multidisciplinary panel using clinical, radiological, operative, and post-mortem evidence where available. ^a^Ventilatory failure includes deaths from airway obstruction, traumatic asphyxia, and tension pneumothorax. ^b^Injuries incompatible with life were defined according to the JRCALC Recognition of Life Extinct (ROLE) criteria (for example decapitation, massive cranial destruction, hemicorporectomy).

Penetrating trauma deaths generally occurred prehospital (137 of 144, 95.1%), in younger patients, and more often male when compared with blunt trauma deaths (*Table 2*). There were also marked differences in the likely cause of death by mechanism (*Fig. 3*): deaths following penetrating trauma were predominantly due to haemorrhage (121 of 144, 84.0%) and included all cases of cardiac tamponade (11 of 144, 7.6%). In contrast, blunt trauma deaths were most often from CNS injury (188 of 353, 53.3%), with a much smaller proportion due to haemorrhage (58 of 353, 16.4%).

**Fig. 3:**
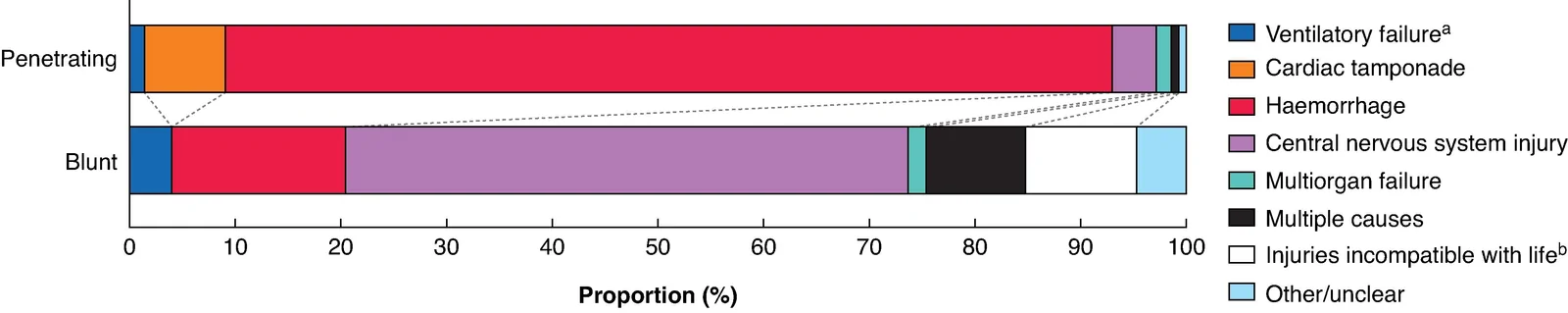
Distribution of the likely cause of death for major trauma patients stratified by mechanism of injury.

^a^Ventilatory failure includes deaths from airway obstruction, traumatic asphyxia, and tension pneumothorax. ^b^Injuries incompatible with life were defined according to the JRCALC Recognition of Life Extinct (ROLE) criteria (for example decapitation, massive cranial destruction, hemicorporectomy).

**Table 2:** Demographic and cause of death differences among 497 trauma deaths by mechanism of injury (blunt *versus* penetrating)

Characteristic	Blunt (*n* = 353)	Penetrating (*n* = 144)	*P*
Age, years	40 (29–60)	26 (20–36)	<0.0001
Male	271 (77.2)	129 (89.6)	0.002
**Phase of care**			
Prehospital	246 (69.7)	137 (95.1)	<0.0001
In-hospital	107 (30.3)	7 (4.9)	<0.0001
**Cause of death**			
Ventilatory failure*	14 (4.0)	2 (1.4)	0.170
Cardiac tamponade	0	11 (7.6)	<0.0001
Haemorrhage	58 (16.4)	121 (84.0)	<0.0001
CNS injury	188 (53.3)	6 (4.2)	<0.0001
Multiple causes	33 (9.4)	1 (0.7)	0.0001
Multiorgan failure	6 (1.7)	2 (1.4)	1.0
Injuries incompatible with life†	37 (10.5)	0	<0.0001
Other/unclear	17 (4.8)	1 (0.7)	0.031

Data are presented as number (%), or median (i.q.r.). CNS, central nervous system. *Ventilatory failure includes deaths from airway obstruction, traumatic asphyxia, and tension pneumothorax. †Injuries incompatible with life were defined according to the JRCALC Recognition of Life Extinct (ROLE) criteria (for example decapitation, massive cranial destruction, hemicorporectomy, or similar massive injuries).

## Discussion

This study provides a comprehensive description of when, where, and why patients die from major trauma within a mature regional trauma system. Deaths followed a highly left-shifted, unimodal distribution. Nearly four-fifths of trauma fatalities occurred before hospital arrival, and almost all traumatic cardiac arrests from potentially reversible causes—haemorrhage, hypoxia, tension pneumothorax, and cardiac tamponade—were prehospital. Deaths following penetrating trauma were likewise almost entirely prehospital, predominantly due to haemorrhage or cardiac tamponade. Together, these findings show that in developed trauma systems, the greatest remaining opportunities to reduce trauma fatalities lie in the prehospital phase of care.

These findings are consistent with prior population-based analyses from mature trauma systems, indicating that most trauma deaths occur early after injury, the ‘consistent curve of death’^2^. They reinforce that haemorrhage and traumatic brain injury remain the two predominant causes of trauma death, together accounting for nearly 80% of fatalities—a pattern remarkably consistent across decades and systems^5,7,8,11,13^. Meanwhile, the absence of a late fatality peak reflects the effectiveness of coordinated, specialist in-hospital care^9,11,15^.

This study extends the literature by defining cause-specific death across phases of care. By capturing deaths occurring before hospital arrival, it addresses the survival bias inherent in hospital-based registries, including the systematic overestimation of the time available for effective haemorrhage control, and demonstrates that the causal profile of trauma deaths differs fundamentally between prehospital and in-hospital phases^2,4^. Similar patterns have been observed in military trauma systems, where improvements in prehospital capability increased overall survival, emphasizing the need for seamless data capture and analyses of deaths across both phases of care^18,19^.

These findings have clear implications for trauma-system design, resource allocation, and public health policy. The predominance of prehospital deaths from exsanguination, particularly following penetrating injury in young patients, highlights the importance of population-level strategies encompassing both primary violence-prevention initiatives and secondary prevention through bystander haemorrhage control^20^. Examples of national haemorrhage-control initiatives aimed at improving training and access to basic bleeding-control equipment include the American College of Surgeons’ *Stop the Bleed* programme in the United States and *CitizenAID* in the UK^21,22^. Furthermore, these data support prioritizing basic haemorrhage-control training and equipment for non-medical emergency first responders, for example police, enabling immediate intervention during the critical early minutes after injury. The narrow window between injury and traumatic cardiac arrest also underscores the importance of effective prehospital capabilities, including early blood product administration and timely treatment of obstructive shock, to prevent traumatic cardiac arrest or enable rapid reversal while still physiologically possible^23–25^. Importantly, this does not imply prolonged on-scene care, but rather the timely delivery of targeted, life-saving interventions alongside rapid transport to definitive hospital care^26^.

The current study is limited by a data set including trauma patients attended by LAA, rather than all trauma deaths in London. However, London’s Air Ambulance is an integral component of the LAS’s major trauma response and is tasked with responding to all suspected major trauma across Greater London 24/7. With dispatch and operational miss rates of approximately 0.5 and 6% respectively, the risk of clinically important selection bias is low, although generalizability to systems with different structures or case mixes may be limited. Second, determination of cause of death, although structured and informed by multiple data sources, involves an element of clinical judgement and may therefore be imperfect in some cases. Third, prehospital deaths were defined by the occurrence of traumatic cardiac arrest before hospital arrival rather than by the point of death declaration; this reflects the physiological threshold beyond which survival after trauma is exceptionally rare and emphasizes that the opportunity for meaningful intervention lies before, or in preventing, cardiac arrest. Fourth, we did not undertake formal case-by-case preventability reviews, so inferences about ‘potentially reversible’ deaths are based on cause-of-death categories rather than individual panel assessment. Finally, this study is descriptive rather than inferential: it identifies where further gains in trauma survival are most likely to be achieved but was not designed to estimate the causal impact of specific interventions or time intervals.

The current study describes when, where, and why patients die from major trauma within a mature regional trauma system. As in-hospital survival rates plateau, further reductions in trauma deaths will require effective, time-critical care closer to the point of injury.

## Supplementary Material

znag030_Supplementary_Data

## Data Availability

Deidentified data underlying this study are available from the corresponding author upon reasonable request, subject to institutional governance approvals.

## Prior presentation

This study was presented in part at the Big Sick Conference, 6 February 2025, Zermatt, Switzerland.
