# Supplementary material for: Epidemiology of trauma deaths across a mature regional trauma system: patterns of prehospital and in-hospital fatalities
Source: Br J Surg. 2026 Mar 18;113(10):znag030. doi: 10.1093/bjs/znag030 (PMC13671401; doi:10.1093/bjs/znag030)
Supplement: Supplementary file 1 [file znag030_supplementary_data.docx]

**Title:** Epidemiology of trauma deaths across a mature regional trauma system: patterns of pre-hospital and in-hospital fatalities.

**Authors:** Aditi Nijhawan^1,2^, Ewoud ter Avest ^1,2,4^, Callum J. Twohig^1,7^,

Stacey J. Webster^1,5^, Jason Morris^1,3^ , Robbie Lendrum^1,2^ , Virginia Fitzpatrick-Swallow ^6^,

David J. Lockey ^1,2,7^, Zane B. Perkins ^1,2,7^

**Affiliations:**

1. London’s Air Ambulance, London, United Kingdom.

2. Barts Health NHS trust, London, United Kingdom.

3. London Ambulance Service NHS Trust, London, United Kingdom

4. University Medical Center Groningen, Groningen, Netherlands.

5. Academic Department of Military Emergency Medicine, Royal Centre for Defence Medicine (Research & Clinical Innovation), Birmingham UK.

6. Home Office Registered Forensic Pathologist, Wantage, United Kingdom.

7. Centre for Trauma Sciences, Queen Mary University of London, London, United Kingdom

**Corresponding Author:**

Dr. Zane Perkins

London’s Air Ambulance

Royal London Hospital 17^th^ floor

London E1 1FR, UK

**ORCID ID:** 0000-0003-4807-8803

**Supplementary Materials - Index**

| **Supplementary Figures and Tables** |  |
| --- | --- |
| Supplementary Table 1 | *page 2* |
| Supplementary Table 2 | *page 3* |
|  |  |

**Supplementary Figures and Tables**

**Supplementary Table 1**: Sources of information informing likely cause-of-death determination in pre-hospital and in-hospital trauma deaths.

| **Source of information** | **Pre-Hospital Deaths**  (n = 383) | **In-Hospital Deaths**  (n= 114) |
| --- | --- | --- |
| Multidisciplinary case review | 383 (100.0) | 114 (100.0) |
| Radiological imaging | 28 (7.3) | 107 (93.9) |
| Operative or procedural findings | 105 (27.4) | 56 (49.1) |
| Post-mortem examination | 110 (28.7) | 8 (7.0) |
| ≥1 supplementary data source available ^a^ | 219 (57.2) | 114 (100.0) |
| Data are presented as number (%).Categories are not mutually exclusive.  ^a^ Availability of at least one radiological, operative or procedural, or post-mortem data source. | | |

Likely cause of death was determined by a multidisciplinary case review using all available information. Clinical assessment and injury pattern were available for all cases and were sufficient where injuries were unequivocal. Radiological imaging, operative or procedural findings, and post-mortem examination were incorporated when available or clinically indicated.

**Supplementary Table 2**: Clinical findings used to estimate the likely cause of death in 164 pre-hospital trauma patients where determination relied on clinical assessment and injury pattern alone.

| **Likely cause of death** | **n (%)** |
| --- | --- |
| Injuries incompatible with life (ROLE criteria) | 37 (22.6) |
| Cranial and cerebral destruction | 17 |
| Decapitation | 3 |
| Hemicorporectomy | 9 |
| Catastrophic torso injury | 8 |
| Ventilatory failure | 12 (7.3) |
| Major airway injury with obstruction | 2 |
| Tension pneumothorax | 7 |
| Traumatic asphyxia | 3 |
| Haemorrhage | 16 (9.8) |
| Penetrating wound in proximity of a major vessel with external exsanguination | 4 |
| Major limb amputation(s) with external exsanguination | 3 |
| Major torso injuries/fractures with signs of exsanguination | 9 |
| Central nervous system injury | 63 (38.4) |
| Penetrating head injury | 4 |
| Isolated head injury | 11 |
| Open skull fracture / extensive cranial injury | 30 |
| Depressed skull fracture/Base of Skull fracture | 12 |
| Head (atlanto-occipital) dislocation | 6 |
| Multiple contributing causes | 23 (14.0) |
| Unclear | 13 (7.9) |
